# Methodology and content for the design of basketball coach education programs: a systematic review

**DOI:** 10.3389/fspor.2025.1614186

**Published:** 2025-06-27

**Authors:** Josep Alemany-Iturriaga, Julio Calleja-González, Jeisson Mosquera-Maturana, Álvaro Velarde-Sotres

**Affiliations:** ^1^ Facultad de Ciencias Sociales y Humanidades, Universidad Europea del Atlántico, Santander, Spain; ^2^ Departamento de Ciencias de Lenguaje, Educación y Comunicaciones, Universidad Internacional Iberoamericana, Arecibo, PR, United States; ^3^ Universidad de La Romana, La Romana, Dominican Republic; ^4^ Department of Physical Education and Sport, Faculty of Education and Sport, University of the Basque Country (UPV/EHU), Vitoria, Spain; ^5^ Facultad de Ciencias de la Salud, Universidad Europea del Atlántico, Santander, Spain; ^6^ Departamento de Salud, Universidad Internacional Iberoamericana, Campeche, Mexico; ^7^ Faculdade de Ciências de Saúde, Universidade Internacional do Cuanza Bairro Kaluanda, Cuito, Angola

**Keywords:** sport, training, basketball, methodology, e-learning, content, education

## Abstract

**Background:**

The increasing complexity of basketball and the need for optimal decision-making in order to maximize competitive performance highlight the necessity of specialized training for basketball coaches. This systematic review aims to compile, synthesize, and integrate international research published in specialized journals on the training of basketball coaches and students, examining their characteristics and needs. Specifically, it analyzes the content, technical-tactical actions, and methodologies used in practice and education programs to determine which essential parameters for their technical and tactical development.

**Methods:**

A structured search was carried out following the Preferred Reporting Items for Systematic Review and Meta-Analyses (PRISMA®) guidelines and the PICOS® model until January 30, 2025, in the MEDLINE/PubMed, Web of Science (WOS), ScienceDirect, Cochrane Library, SciELO, EMBASE, SPORTDiscus, and Scopus databases. The risk of bias was assessed and the PEDro scale was used to analyze methodological quality.

**Results:**

A total of 14,090 articles were obtained in the initial search. After inclusion and exclusion criteria, the final sample was 23 articles. These studies maintained a high standard of quality. This revealed data on the technical-tactical actions addressed in different categories; the profiles, characteristics, and influence of coaches on player development; and the approaches, teaching methods, and evaluation methodologies used in acquiring knowledge and competencies for the professional development of basketball coaches.

**Conclusions:**

Adequate theoretical and practical training for basketball coaches is essential for player development. Therefore, training programs for basketball coaches must integrate technical-tactical, physical, and psychological knowledge with the acquisition of skills and competencies that are refined through practice. This training should be continuous, more specialized, and comprehensive, focusing on understanding and constructing knowledge that supports the professional growth of basketballers. Additionally, training should incorporate digital tools and informal learning opportunities, with blended learning emerging as the most effective methodology for this purpose.

**Systematic Review Registration:**

https://www.crd.york.ac.uk/PROSPERO/view/CRD420251000058, PROSPERO CRD420251000058

## 1 Introduction

In recent years, there has been a lot of academic research on sport performance, with a focus on identifying and understanding the various factors that contribute to athletic success (1). Thus, in the field of sport coaching, several stakeholders contribute to an athlete's development, most notably coaches, who have a significant influence on athletes' performance levels at all stages of their sporting lives (2), playing a particularly important role in providing support in all domains, including the emotional dimension (3).

To effectively fulfill their roles within the evolving paradigm of lifelong learning (4), sports coaches must receive continuous and comprehensive training. This enables them to carry out their educational responsibilities (5), utilizing all available knowledge and coaching methods to guide athletes throughout their careers (6). Such training is fundamental not only for fostering positive sports experiences (7) and athletic achievement (8) but also for contributing to the social and human capital of nations (9). As a result, many countries have established specific structural and organizational frameworks for the sports sector (10).

In that way, new sports training models have emerged that emphasize understanding and knowledge construction, offering diverse perspectives on human capability development (11). These models shape contemporary sports training across all forms of modern sport, including Olympic, recreational, and professional sports (12). To implement these models effectively, coaches require both theoretical knowledge and practical application skills (13) in order to promote physical fitness, teamwork, and leadership development (14). In this regard, Mesquita et al. (15) highlight the need for coach education curricula that adopt an in-depth and accessible learning approach, integrating comprehensive instruction in technique and tactics. This involves identifying the key components of such teaching (16).

Thus, sports federations and universities offer training programs for coaches, since they are the educational institutions responsible for teaching and training sports coaches with official technical training courses and courses related to sports science (17).

In particular, basketball presents a unique case. According to the International Basketball Federation (18), approximately 610 million people play basketball in some capacity, making it one of the world's most popular sports (19), ranking behind only soccer, swimming, and volleyball. Moreover, basketball coaching has become an important sector with the development of the internet and social media (20), which underlines the need for a specialized approach to basketball coach education (21), especially at the present time, when there is a growing demand for innovative training models created from emerging pedagogies, including e-learning based methodologies (22), as the newest and most popular form of distance education today (23). While national federations often develop training programs to enhance coaching quality and knowledge in their respective countries (17), there is a growing demand for training that addresses not only the increasing number of participants but also the complexities of modern basketball (24), as well as training contents, curricular pedagogy, objectives, strategies, types of training (25), and the development of basketball coach competences, stimulating training that covers all types of coaches, from those concerned with player training to those dedicated to high performance (21). In that sense, coaches must be equipped to make informed decisions that maximize competitive performance (26). A training that overcomes traditional face-to-face models based on conferences, lectures and training sessions in sports facilities (27). Heterogeneous training models, with different academic, pedagogical, and methodological criteria around the world, as a consequence of the existence of different regulations depending on the specific structure of the sport and the sport branch in each country (9). Training that ensures that students acquire the necessary knowledge and skills in an effective and meaningful way through online sports training, which, according to Reyero (28), must integrate ICT in a constructivist methodology that motivates, teaches to think and learn. And in the case of basketball coaches, it should adequately train them in Motivation Competency, Game-strategy competency, Character-building competency and Technique competency (29).

However, to date, and to the best of our knowledge, there are no previous scientific studies that allow us to design a program that meets the training needs of basketball coaches in a homogeneous manner. Therefore, the main objective of this systematic review is to compile, synthesize, and integrate international research published in all scientific databases related to the methodologies and content used in designing training programs for basketball coaches. This study follows the research direction of Leite et al. (21, 30), aiming to identify the essential components of technical and tactical training for basketball coaches.

## 2 Methods

### 2.1 Searching strategies

This article presents a systematic review focused on identifying the essential methodologies and content for the technical and tactical training of basketball coaches. This systematic review was conducted following the Preferred Reporting Items for Systematic Reviews and Meta-Analyses (PRISMA®) guidelines (23), ensuring completeness. It was registered in PROSPERO (ID = CDR420251000058). Methodological issues were resolved with guidance from the Cochrane Handbook for Systematic Reviews of Interventions (31).

The PICOS® model was used to determine the inclusion criteria (32): P (Population): «coach», «basketball»; I (Intervention): «training», «education»; C (Comparators): «e-learning», «face-to-face»; OR (Outcome): «contents», «methodology»; and S (study design): «any type of design».

A structured search was performed in MEDLINE/PubMed, Web of Science (WOS), ScienceDirect, Cochrane Library, SciELO, EMBASE, SPORTDiscus, and Scopus. The research ended on January 30, 2025. Search terms included free text words for key concepts related to basketball coaches, basketball players, methodologies, and content worked in basketball. Specifically, the following search equation was used: [«coach» (MeSH Terms) OR «sports coach» OR «basketball coach» OR «sports technician» OR «basketball technician» AND «training» (MeSH Terms) OR «education» AND «e-learning» (MeSH Terms) OR «classroom» AND «content» (MeSH Terms) OR «subjects» AND «methodology» (MeSH Terms) OR «teaching»]. All relevant articles in the field were obtained using this equation. The reference sections of all identified articles were also examined by applying the «snowball method» strategy (33), based on the examination of the reference sections of the identified articles. the scientific literature review and controlled vocabulary such as Medical Subject Headings (MeSH). The search was not restricted by publication date but was filtered to include only English-language studies in humans. The search was not restricted by publication date but was filtered to retrieve only human studies written in English. The last search was conducted on 30 JAN 2025. All search titles and abstracts were cross-checked to identify duplicates and any potential missing studies (J.A.-I. and A.V.-S.). Titles and abstracts were selected for later review of the full text. The search for published studies was conducted independently by two different authors (J.A.-I. and Á.V.-S.), and disagreements were resolved by discussions between them.

### 2.2 Inclusion and exclusion criteria

Studies were selected based on their provision of information on the methodologies used in training basketball coaches and students, including their profiles, educational and experiential levels, competencies, training content, and game situations. Original studies related to basketball were included in the systematic review, while systematic reviews, meta-analyses, conference abstracts, and opinion articles were excluded. Additionally, only studies with a minimum of 10 participants were selected.

For the articles retrieved in the search, the following inclusion criteria were applied to the final selection of studies: (I) studies less than 15 years old; (II) studies with a minimum sample of 10 participants; (III) original articles related to basketball; (IV) studies on the education of sports coaches; (V) studies on online learning; and (VI) studies on face-to-face learning. The following exclusion criteria were applied to the experimental research protocols: (I) systematic reviews; (II) studies more than 15 years old; (III) studies with less than 10 participants; (IV) studies not related to basketball; (V) studies not related to sports coach education.

Based on the scientific studies on sports and basketball analyzed, these inclusion and exclusion criteria were chosen with the objective of compiling original basketball studies that included a minimum of 10 participants, coinciding with the minimum number of participants in similar scientific studies; and that they included either face-to-face or e-learning training for basketball coaches and trainers and basketball students in both formal and non-formal education settings.

### 2.3 Study selection

Titles and abstracts of publications identified by the search strategy were selected for subsequent full-text review and cross-referenced to identify duplicates (889). All trials assessed for eligibility and classified as relevant were retrieved, and the full text was peer-reviewed (J.A.-I. and A.V.-S.). In addition, the references section of all relevant articles was also examined using the snowball strategy (33). Based on the information in the full articles, inclusion and exclusion criteria were used to select eligible trials for inclusion in this systematic review. Disagreements were resolved by discussions between two authors (J.A.-I. and A.V.-S.).

### 2.4 Data extraction

Once the inclusion and exclusion criteria were applied to each study, the following data were extracted: study source [author(s) and year of publication], sample population (including the number of participants or technical-tactical actions), competitive level or experience, research design type, study variables, main results, and effects of the intervention.

For each study, information was carefully collected from all eligible publications. Mean (±), standard deviation (SD), and sample size data were extracted from the tables of all included papers. Any disagreements were subsequently resolved through discussion until a consensus was reached (J.A.-I. and A.V.-S.).

### 2.5 Quality assessment and risk of bias

Methodological quality and risk of bias were assessed by two authors independently (J.A.-I. and A.V.-S.), and disagreements were resolved by assessment by a third party (J.C.-G.), in accordance with the Cochrane Collaboration Guidelines (31).

The Cochrane risk of bias tool was used to assess bias, with the following items categorized into different domains: (1) selection bias (items, random sequence generation, allocation, and concealment), (2) performance bias (blinding of participants and staff), (3) detection bias (blinding of outcome assessment), (4) attrition bias (incomplete outcome data), (5) information bias (selective information), and (6) other biases (other sources of bias).

For each study, the risk of bias was classified as «low» if the criteria indicated a low risk of bias (unlikely to significantly affect results) or «high» if the criteria suggested a high risk of bias (potentially undermining the reliability of the results). If the risk of bias was uncertain, it was categorized as «unclear» (raising doubts about the findings).

This systematic review followed the principles outlined in the PRISMA® statement (34), a checklist designed to enhance transparency in systematic reviews and ensure the scientific rigor of these studies. PRISMA® includes 27 items and a four-stage flowchart, outlining essential components for the clear and systematic reporting of research findings.

The “Physiotherapy Evidence Database (PEDro)” scale was also used to analyze the methodological quality of all the selected articles. This scale is a tool designed to evaluate the methodological quality of the clinical designs (Table 1) and used in many bibliographic reviews. The aforementioned tool is based on a list developed by Verhagen et al. (35) using the Delphi technique.

**Table 1:** “Physiotherapy evidence database (PEDro)” scale to analyse the methodological quality of the studies.

PEDro scale			
1	The criteria of election were specified	Yes	No
2	The subjects were randomly assigned to the groups	Yes	No
3	The assignment was hidden	Yes	No
4	The groups were similar at the beginning in relation to the indicators of prognosis	Yes	No
5	All subjects were blinded	Yes	No
6	All the sports scientists providing therapy were blinded	Yes	No
7	All assessors evaluating at least one of the key results were blinded	Yes	No
8	All the measures of at least one of the key results were obtained from more than 85% of the subjects initially assigned to the groups	Yes	No
9	The results of all the subjects receiving treatment or assigned to the control group were given, or when not possible, the data for at least one key result were analysed “in order to treat”	Yes	No
10	The results of statistic comparisons among groups were reported for at least one key result	Yes	No
11	The study provides specific and variability measures for at least one key result	Yes	No

The PEDro scale has a total of 11 items. Item 1 refers to the external validity of the study, while items 2–9 refer to the internal validity; items 10 and 11 show if the statistical information provided by the authors allows the accurate interpretation of the results. All items in the list are dichotomized as “yes”, “no” or “not reported”. Each “yes” item is given one point, while “no” or “not reported” items do not receive any points at all.

The first item of the PEDro scale was not considered in this review, as it was related to the evaluation of the external validity of the studies. Therefore, only items 2–11 were selected for the assessment of the methodological quality. Due to this, the maximum score of an article could not be higher than 10 points, and the minimum could not be lower than 0 points.

The evaluation of the heterogeneity was another point to analyze. In this case, we can consider, on the one hand, clinical heterogeneity, due to the differences among the types of patients, treatments, and endings, and on the other hand, methodological heterogeneity, due to the variability in the designs and bias control.

## 3 Results

### 3.1 Main research

The database search initially identified 14,090 publications, with 889 excluded as duplicates. After a detailed review of titles, abstracts, and full articles, 52 publications met the inclusion criteria. A publication timeframe of 15 years was applied, narrowing the selection to 34 publications eligible for evaluation. In the final stage of article inclusion, 8 studies that did not report research results and 3 studies with fewer than 10 participants were excluded.

**Figure 1:**
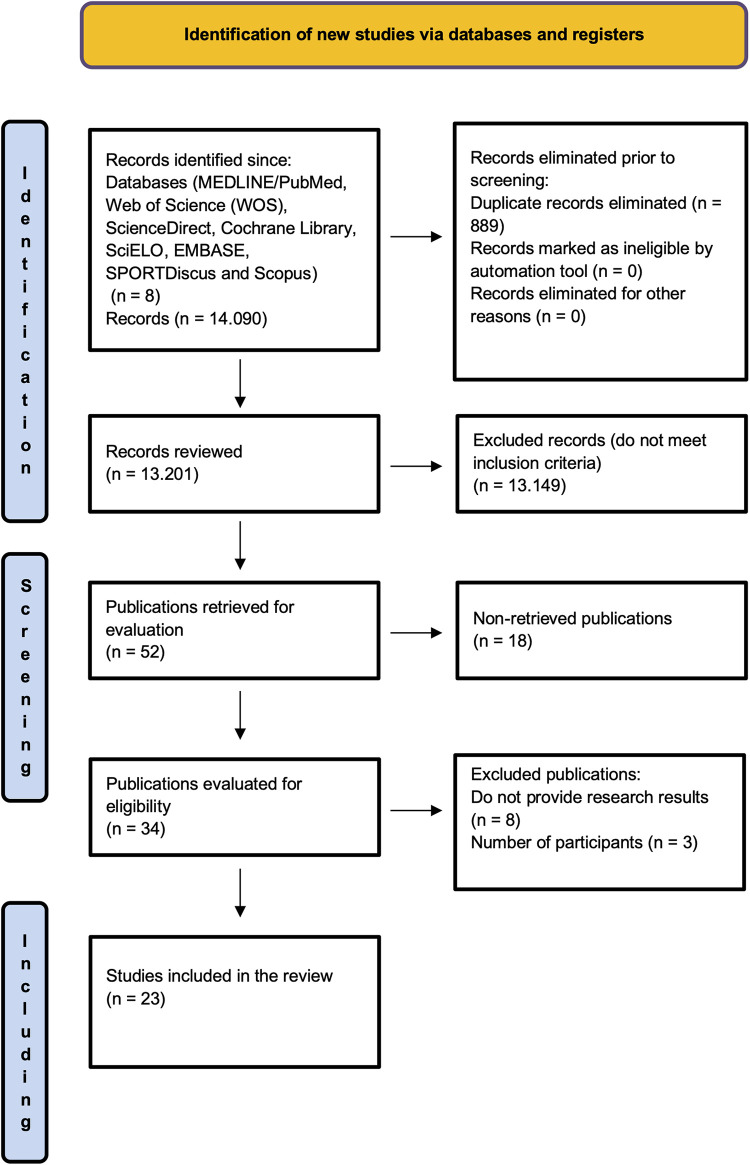
Flow diagram of the study selection.

Of the final selection, 23 studies were included. 8 articles (21, 25, 30, 36–40) with significant data concerning contents, technical-tactical actions, priorities, and approaches in coach education according to level and category. 3 articles (41, 42, 54) with significant data concerning the profile, characteristics, and influence of coaches. 7 articles (22, 43–48) with significant data concerning basketball coaches' competencies, knowledge, and professional development. 5 articles (49–53) with significant data concerning teaching and evaluation methods (Figure 1).

### 3.2 Study characteristics

This study is a systematic review of original research analyzing the content and methodologies used in designing training programs for basketball coaches and students. A total of 23 articles containing significant data in this field were included, based on factors such as profile, educational and experiential level, competencies, training content, and game situations. Basketball-related studies were selected if they had a minimum of 10 participants and were published within the past 15 years.

The selected articles were categorized based on the sample population, resulting in 4 subgroups: basketball coaches, basketball players, basketball students, and technical-tactical actions. The key dependent variables examined in this study included the methodologies used in basketball coach training, training session content, game situations and competencies, and the profile, educational background, and experience level of coaches.

The main results and effects of the intervention are shown in Table 2.

**Table 2:** Methodology and results of the interventions*.*

Authors	Sample	Level	Design	Variables	Results	Effects
Basketball coaches						
Leite et al. (21)	185 basketball coaches.	Regional competitions and national teams.	Descriptive and cross-sectional.	Technical Fundamentals. Tactical Fundamentals. Physical Fundamentals. Exercise Characteristics.	Greater emphasis on basic defensive movements by coaches working with players up to 14 years. In men greater focus on conditional capacities; women on coordinative capacities. Significant differences in the importance given to the duration, decision-making, and the recreational component.	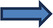
Alemany et al. (22)	50 basketball coaches (34 men and 14 women).	Regional competitions and regional teams.	Quantitative, non-experimental, and cross-sectional.	Training modality: E-learning vs. face-to-face. Demographics: age, gender, place of residence. Skills acquisition.	24.60% of e-learning participants felt “fully qualified” as basketball coaches, compared to 10.98% of face-to-face trained coaches. E-learning training effectively qualifies basketball coaches in the Cantabria region.	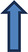
Junior et al. (42)	94 basketball coaches with a minimum of one year of experience.	Inexperienced, national, and international coaches.	Descriptive and comparative.	Coach level. Factors influencing basketball talent: anthropometric, physical, technical, tactical, psychological, and contextual.	International-level coaches place more emphasis on the anthropometric and contextual factors than others. Physical factors are valued by all coaches, regardless of level. Technical, tactical, and psychological factors are considered more critical for point guards than other positions.	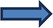
Moletta et al. (44)	19 basketball coaches.	State Championship.	Qualitative and descriptive.	Types of coach learning: Formal (academic background), non-formal (training courses and activities), and informal (professional experience and knowledge sources).	Most coaches have a background in physical education and participated in both face-to-face and online training activities, exchanging knowledge with peers, reading books and articles, and drawing from experience as players or assistant coaches.	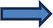
Leite et al. (30)	212 basketball coaches.	Level 1 (Initiation). Level 2 (Intermediate). Level 3 (Expert).	Descriptive and cross-sectional.	Stage of sports training. Gender of coached team. Training content is grouped into four dimensions: technical, tactical, physical, and psychological.	Initiation-level coaches emphasize individual technical and tactical content. Intermediate-level coaches prioritize more complex technical and tactical skills. Expert-level coaches focus on collective tactical elements and specific physical preparation. Coaches give greater importance to technical and tactical content over physical and psychological content.	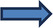
Guedea-Delgado et al. (43)	951 basketball coaches (513 women and 438 men).	National coaches	Quantitative, non-experimental, descriptive and cross-sectional.	Coach characteristics. Self-assessed knowledge in seven areas: sports training, physiology, tactics, psychology, biomechanics, management and research.	Majority of coaches feel their knowledge of training planning and cycles; basketball management and psychological knowledge is insufficient. 37.2% report a limited variety of exercises and express a desire to learn more. 59% rarely or never analyze training methodologies or attend professional development courses.	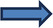
Herrera-López et al. (37)	117 basketball coaches	U14-U16 teams	Quantitative descriptive	Coach's profile. Education and training. Training contents. Training methodology.	The majority of coaches are male, aged 25–44, with level 2 training, most of them former basketball players. 49.6% consider the training received insufficient and prioritize technical preparation over tactical and physical aspects. Lack of structured progression in training content.	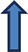
Tan & O’Connor (45)	124 Level 1 coaches with at least 10 years of experience and registered with NROC for at least 5 years.	Singapore National Register of Coaches Level 1.	Mixed with an explanatory sequential approach.	Coaches’ knowledge: intrapersonal, interpersonal, and professional. Learning needs, preferences for learning sources, barriers, and difficulties. Motivations to participate in continuing education. Value placed on player development.	Intrapersonal knowledge was highly valued. Main learning needs included sport psychology, use of technology in training, and career planning and development. The main motivations were having a license and the relevance of the content. The main barriers were time and cost.	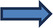
Jiménez (41)	89 basketball coaches (72 men and 17 women)	U12–U14 teams in the Community of Madrid.	Descriptive.	Coach profile. Perception of decision-making in players: perceived importance and conceptual definitions provided by coaches. Training activities and strategies used to improve decision-making.	Decision-making is considered fundamental to player development; some coaches linked it to autonomy and freedom, while others focused on reading the game and stimulus perception. Few coaches used questioning feedback or post-task reflection. The study highlights the need for ongoing training in tactical teaching at early stages.	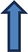
Ortega (36)	102 basketball coaches	Level 1 Level 2 Level 3	Descriptive.	Basic Collective Technical-Tactical (CTBMs): passing and progression, direct and indirect blocking, Complex collective technical-tactical (MTCCs): fastbreak, defensive balance, positional attack, type of defense.	Passing and progression is emphasized in U14 categories, while blocking becomes more important at U18 categories. Fastbreak is the most highly valued action at all levels, while individual collective defense and defensive balance are considered more important than positional attack. Zonal defense is introduced from U16 onwards.	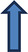
Ciampolini et al. (46)	68 basketball coaches and four basketball monitors.	Level I and Level II.	Descriptive with mixed approach.	Curricular structure of the program. Teaching strategies used in the program. Coaches’ perception of the program.	93% of coaches expressed satisfaction with the program, highlighting the diversity of approaches and the practical applicability of the content as key strengths. Lack of practical sessions and a need for more content on refereeing and sports management.	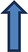
Balogh & Trzaskoma-Bicsérdy (54)	152 basketball coaches (105 men and 47 women) with an average experience of 12.9 years (±11.5.)	Level 1 Level 2 Level 3	Descriptive and comparative.	Pedagogical and professional roles. Personal characteristics. Attitudes and behaviors.	Coaches over 55 emphasized teaching values and motivating players with patience and use punishment as a disciplinary method. Younger coaches value less the educational role of the coach and focus more on tactics and performance. All coaches strongly rejected verbal or physical abuse and inappropriate language.	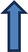
Mendes et al. (47)	20 coaches	Paraná state	Cross-sectional descriptive.	Sources of knowledge used by coaches. Relationship between the sources of knowledge and the level of academic degree, the length of professional experience and the competitive level of the teams being trained.	Academic education is the most frequently used source of knowledge by coaches (8.34 ± 1.25), followed by professional experience (7.63 ± 1.87). Coaches with higher academic degrees rely less on professional experience and experience as players, while those with less academic education rely more on their experience. Coaches at higher competitive levels place greater emphasis on academic education.	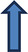
Basketball players						
Kao et al. (48)	438 basketball players (251 male and 187 female)	Divisions I and II of men's and women's college basketball in Taiwan.	Quantitative, non-experimental and cross-sectional.	Coaching competencies. Confidence in the coach	Assessment of the four dimensions of competence—motivation, game strategy, technique and character development—positively predicts players’ confidence in their coach. Improving coaches“ psychological and tactical skills, their ability to identify talent and their instructional effectiveness, along with fostering a positive attitude towards sport, may contribute to players” increased trust in their coaches.	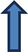
Reina et al. (38)	12 basketball players	U 14 girls team	Quantitative, descriptive and cross-sectional.	Game situation Phase of play Exercises and tactical situations.	Unopposed tasks were related to attack training and individual technical skills. Reduced situations with numerical equality were related to attack training, while reduced situations with numerical imbalance were related to defensive training. Real game situations (5 × 5) were associated with collective tactical behaviors.	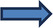
Basketball students						
Riza et al. (53)	30 university basketball students	University	Research and development with a mixed approach.	Basketball learning model based on blended learning. Student learning autonomy. Effectiveness of the learning model.	The learning model developed was validated and rated as “very valid” and “very good”. The implementation of the model led to a significant improvement in students’ learning autonomy in the context of basketball education, reflected in a 27.08% increase in the average score.	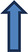
Liu and Wang (51)	30 students	University	Descriptive and analytical	Type of university and level of experience of professors. Assessment methods. Content and frequency of assessment, weight of different components in the final grade. Teacher and student perceptions of assessment practices.	Most universities use a combination of formative and summative assessment methods, with written examinations being the predominant assessment method. More experienced teachers tend to apply a wider variety of assessment methods. The assessment system should better reflect the competences required in basketball coaching.	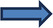
Papastergiou and Gerodimos (52)	88 high school students (45 in mixed mode and 43 in face-to-face)	University	Experimental with pretest/post-test design	Basketball knowledge. Acceptance of the web course.	Both groups improved their knowledge. The combination of web learning and face-to-face instruction proved to be more effective than traditional teaching alone.	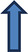
González-Espinosa (49)	40 students	Primary education	Longitudinal quasi-experimental	Teaching method. Technical skills. Technical Execution. Performance indicators. Efficiency Performance.	The alternative method (PEAB) showed significantly greater improvements in decision making (*p* ≤ .01), performance efficiency (*p* ≤ .05) and overall performance (*p* ≤ .05). Students in the alternative group demonstrated better decision-making.	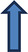
Technical-tactical actions						
González-Espinosa (50)	40 training tasks	Scholar sport	Instrumental	Pedagogical variables. External load variables.	In direct Instruction (DIB) unopposed tasks predominate, while the Tactical Game approach (TGB) incorporates real opposition. While both methods are valid, TGB is more effective for developing tactical skills, decision-making, and meaningful learning.	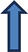
Cañadas (25, 55)	783 technical-tactical actions	U12-U14 teams	Empirical qualitative	Game situations. Comparison between U12 and U14 teams.	In the U12 teams 1 × 1 situations predominate (39.85%), followed by 2 × 2 (16.24%) and 3 × 3 (10.41%). In U14 teams, most tasks are 1 × 1 (42.53%), followed by 5 × 5 (14.94%) with a model that mainly emphasizes technical instruction.	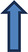
Agüera-Maturana et al. (39)	352 technical-tactical actions of 1 × 1 and 41 male basketball players.	U14 team	Observational and descriptive	Start of the 1 × 1. Development of 1 × 1. Completion of 1 × 1.	The 1 × 1 is most often initiated from the outside areas. The right side exit is the most commonly used, but the center exit is more effective. The most commonly used passing path is from inside to outside, but passing from outside to inside is more effective. The restricted area is the most commonly used and also the most effective finishing area, along with the three-point shot.	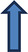
Moreno-Ariza et al. (40)	100 technical-tactical actions distributed over 20 sessions.	U14 team	Empirical, quantitative and descriptive	Pedagogical variables. External load variables. Organizational variables.	Numerical equality tasks are the most used (72%), with high-intensity tasks predominating (68%). Small-sided games are the main method of teaching tactics. There are more tasks focused on attack (45%) than on defense (40%). Player participation is high, with 81–100% active participation in most tasks. Tasks involving larger groups increase the cognitive load and decision-making demands.	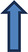

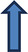
 Positive effect; 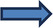 No effect; 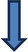 Negative effect.

### 3.3 Risk of bias

Methodological quality and risk of bias were assessed according to the Cochrane Collaboration guidelines (31).

**Figure 2:**
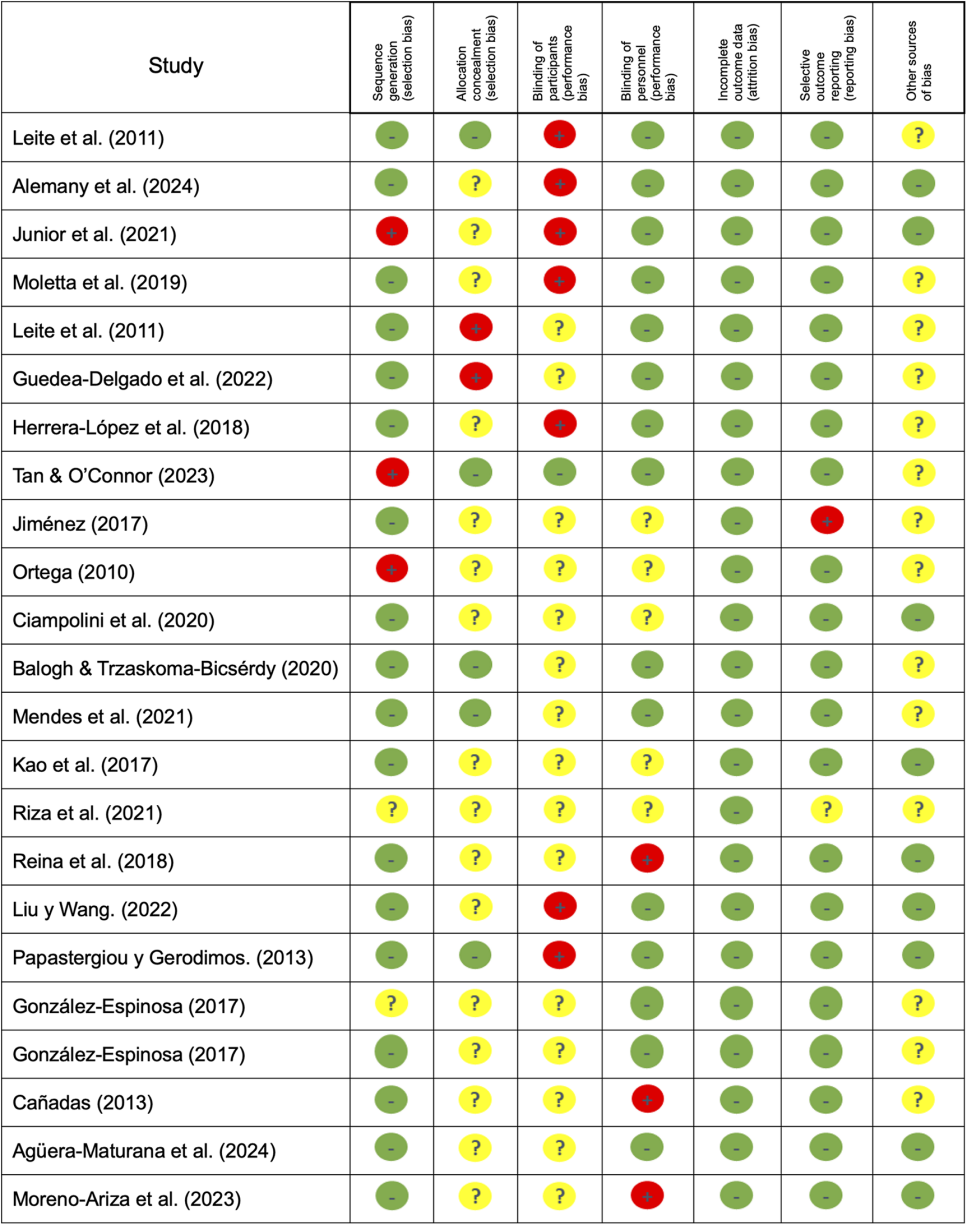
Risk of bias summary: review of author's judgments about each risk of bias item presented as percentages across all included studies.

**Figure 3:**
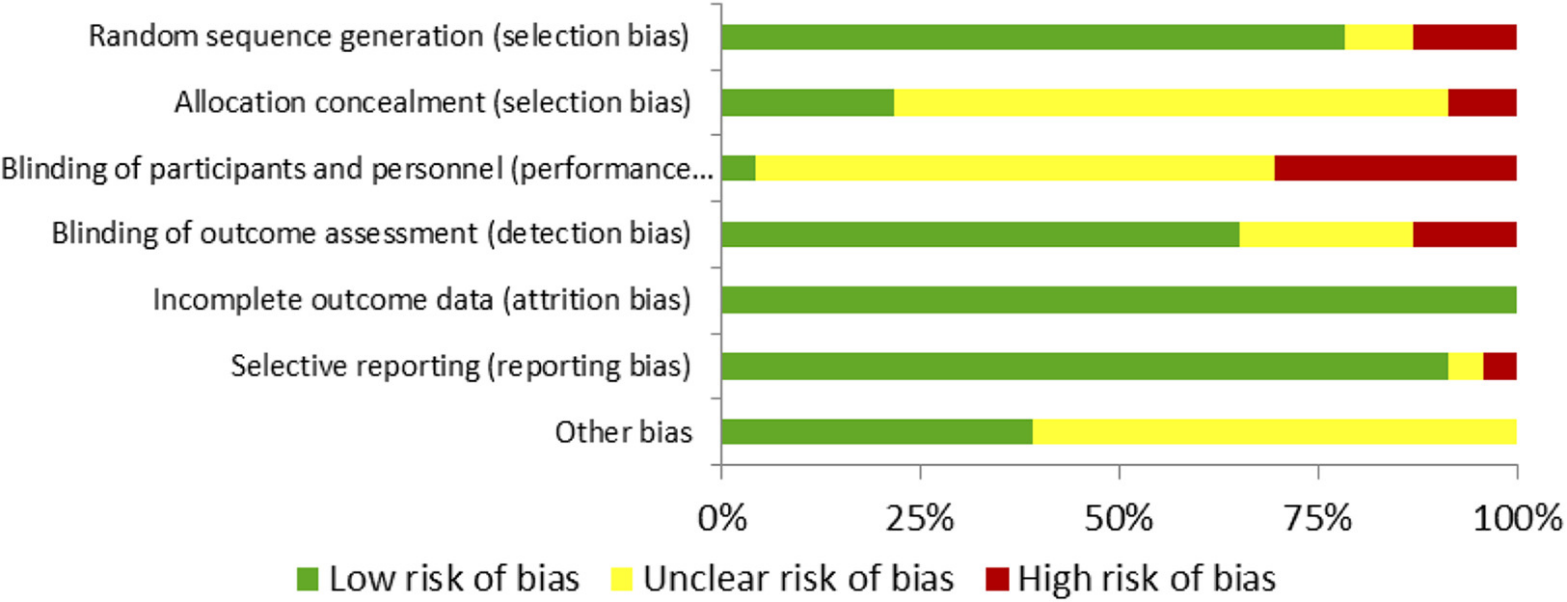
Risk of bias graph: review of author's judgments about each risk of bias item for each included study.

For each study, bias was classified as «low» if the criteria were met for low risk of bias (unlikely to seriously alter the results) or «high» if the criteria were high risk of bias (seriously undermines the reliability of the results). If the risk of bias was uncertain, it was categorized as «unclear» (raising doubts about the findings). The risk of bias of each included study was assessed (31). The complete quality assessments of the studies are shown in Figures 2, 3.

### 3.4 Methodological quality assessment

The methodological quality of the analyzed studies varied between 2 and 8 points, with an average of 5.52 points. 13 articles got 6 points, 7 articles got 5 points, 1 article got 8 points, 1 article got 4 points and 1 article got 2 points. Most of them fulfilled item 2, called “The subjects were randomly assigned to the groups”; item 8, called “All the measures of at least one of the key results were obtained from more than 85% of the subjects initially assigned to the groups”; item 9, called “The results of all the subjects receiving treatment or assigned to the control group were given, or when not possible, the data for at least one key result were analysed in order to treat”; and item 10, “The results of statistic comparisons among groups were reported for at least one key result”. All of them fulfilled item 11, called “The study provides specific and variability measures for at least one key result”. Most of them did not comply with item 3, called “The assignment was hidden”, and item 5, called “All subjects were blinded”. All of them did not comply with item 4, “The groups were similar at the beginning in relation to the indicators of prognosis”, item 6, called “All the sports scientists providing therapy were blinded”, and item 7, called “All assessors evaluating at least one of the key results were blinded” (Table 3).

**Table 3:** Results according to PEDro scale (*n* = 23).

Clinical trial	1	2	3	4	5	6	7	8	9	10	11	TOTAL
Leite et al. (21, 30)	YES	YES	NO	NO	NO	NO	NO	YES	YES	YES	YES	6
Alemany et al. (22)	YES	NO	NO	NO	NO	NO	NO	YES	YES	YES	YES	5
Junior et al. (42)	YES	NO	NO	NO	NO	NO	NO	YES	YES	YES	YES	5
Moletta et al. (44)	YES	YES	NO	NO	NO	NO	NO	YES	YES	YES	YES	6
Leite et al. (21, 30)	YES	YES	YES	NO	YES	NO	NO	YES	YES	YES	YES	8
Guedea-Delgado et al. (43)	YES	YES	NO	NO	NO	NO	NO	YES	YES	NO	YES	5
Herrera-López et al. (37)	YES	YES	NO	NO	NO	NO	NO	YES	YES	YES	YES	6
Tan & O’Connor (45)	YES	YES	NO	NO	NO	NO	NO	YES	YES	YES	YES	6
Jiménez (41)	YES	YES	NO	NO	NO	NO	NO	YES	YES	YES	YES	6
Ortega (36)	YES	NO	NO	NO	NO	NO	NO	YES	YES	YES	YES	5
Ciampolini et al. (46)	YES	NO	NO	NO	NO	NO	NO	YES	YES	YES	YES	5
Balogh & Trzaskoma-Bicsérdy (54)	YES	YES	NO	NO	NO	NO	NO	YES	YES	YES	YES	6
Mendes et al. (47)	YES	YES	NO	NO	NO	NO	NO	YES	YES	YES	YES	6
Kao et al. (48)	YES	YES	NO	NO	NO	NO	NO	YES	YES	YES	YES	6
Riza et al. (53)	YES	YES	NO	NO	NO	NO	NO	YES	YES	YES	YES	6
Reina et al. (38)	YES	NO	NO	NO	NO	NO	NO	YES	YES	YES	YES	5
Liu y Wang. (51)	YES	YES	NO	NO	NO	NO	NO	YES	YES	YES	YES	6
Papastergiou y Gerodimos (52)	YES	YES	NO	NO	NO	NO	NO	YES	YES	YES	YES	6
González-Espinosa (49)	NO	NO	NO	NO	NO	NO	NO	NO	NO	YES	YES	2
González-Espinosa (50)	YES	YES	NO	NO	NO	NO	NO	YES	YES	YES	YES	6
Cañadas (25, 55)	NO	NO	NO	NO	NO	NO	NO	YES	YES	YES	YES	4
Agüera-Maturana et al. (39)	NO	YES	NO	NO	NO	NO	NO	YES	YES	YES	YES	5
Moreno-Ariza et al. (40)	YES	YES	NO	NO	NO	NO	NO	YES	YES	YES	YES	6

## 4 Discussion

### 4.1 Summary of main findings

The primary objective of this systematic review is to compile, synthesize, and integrate international research published in various scientific databases on the methodologies and content used in designing basketball coach training programs. 23 studies were used in the review on content in basketball coach training.

This review highlights the variability in prioritized content based on the level and category of both coaches and players, reinforcing the importance of maintaining a structured and coherent progression in the teaching-learning process throughout an athlete's development. As noted by Delmas (56), failing to do so may hinder the holistic development of players in the long term, a conclusion also supported by Herrera-López et al. (37).

To ensure the professional growth of basketball coaches, continuous training in various contexts is recommended. This approach should integrate formal academic education (47) with practical experience and informal learning (44), fostering a well-rounded coaching education (57).

Research indicates that active and contextualized methods are more effective than traditional approaches (49, 50). Blended learning has been identified as the most effective training option for coaches (58), particularly due to its ability to enhance autonomy in the learning process (52, 53). Assessment is a crucial component of this process (59), requiring diversification of evaluative methods beyond traditional written exams to ensure comprehensive training (51, 60).

When designing training programs, it is essential to consider the profile and characteristics of basketball coaches, including their level and experience (21). This aligns with modern methodological trends in sports training, where coaches are no longer solely responsible for technical, tactical, and physical development but also play a vital role in addressing psychological and emotional aspects (61). Based on the findings of this review, it is recommended to incorporate areas such as decision-making and player feedback (41). Additionally, other key factors for maximizing performance—such as self-awareness and emotional management—should be emphasized, as they significantly influence coaching approaches (26, 45).

### 4.2 Basketball coaches

The competence of a basketball coach is defined by technical-tactical knowledge, communication skills, and leadership, all of which significantly influence player development, both in terms of sports performance and personal growth (41, 42, 54).

Coaches' experience and training levels vary widely, ranging from inexperienced coaches at the international level to those at the expert and professional levels (21, 42, 46). Early-stage coaches tend to focus on individual technical-tactical aspects, whereas advanced-level coaches emphasize collective tactics and physical preparation (21). Coaches identify gaps in their knowledge, particularly in training planning, work cycles, player specialization by position, psychology, and sports management (43). Additionally, self-awareness and personal development are valued more highly than interpersonal or professional knowledge (45).

While basketball coaches recognize the importance of continuous training and staying updated, they express dissatisfaction with the current training offerings (37). The need for continuous professional development is evident, particularly in emerging trends and technologies, analytical and problem-solving skills, and sport psychology and planning, all of which are essential for adequate professional growth (22, 43–48).

Coaches believe that effective training requires the ability to teach and develop players, set and plan goals, and maintain motivation. They place particular emphasis on ethics, patience, tolerance, and empathy as essential qualities for coaching (54).

### 4.3 Basketball players

Coach education plays a crucial role in player development (22), particularly benefiting coaches who foster positive learning environments through motivation while promoting autonomy and constructive feedback as core principles of a holistic player development process (21, 30, 36–40, 55).

Besides, decision-making is regarded as fundamental to a basketball player's learning process, highlighting the need for problem-solving exercises and small-sided game situations (62). Therefore, training should be adapted to the individual needs of each player, considering their level and age, to effectively develop the skills and competencies relevant to their growth (63).

Finally, coaches prioritize specific offensive and defensive skills, including driving to the basket, shots close to the basket, dribbling and passing, especially counterattack; while in defense, defense to the player with the ball and defensive rebounding stand out (37).

### 4.4 Basketball students

Most basketball studies combine formative and summative assessment, although there is a lack of diversity in assessment methods, with written examination being predominant (51). Students value web courses positively, since, when combined with face-to-face, it allows for more effective teaching than the traditional one (52). Innovative and alternative teaching methods applied to basketball students improve decision-making and performance efficiency.

A comprehensive formative approach is needed, both in formal and informal contexts, attending to the development of technical-tactical skills, social skills, and values, using innovative teaching and assessment methods, integrating ICT that fosters meaningful learning through the active participation of basketball students in real game situations (49–52).

### 4.5 Technical-tactical actions

Among the technical-tactical actions analyzed, the most emphasized include bouncing, shooting, rebounding, counterattacking, individual defense, and collective individual defense (21, 30, 36–40, 55). Dribbling and passing are key in minibasket and children's categories, whereas blocking and zone defense gain importance at the cadet and junior levels (36). The counterattack is the most valued offensive action, while odd fixation is the most valued defensive action, across all age categories. Collective individual defense and defensive balance are prioritized over positional attack (36).

Then, mastering these technical-tactical skills, combined with effective decision-making in dynamic game situations, is essential for developing successful basketball players (21, 41, 42).

### 4.6 Strengths, limitations, and future research directions

This study effectively compiles and analyzes key research on the training of basketball coaches and students, highlighting the content covered and methodologies used in basketball training. Additionally, this systematic review identifies trends and essential elements in basketball coach education—one of the most widely practiced sports globally—while adhering to PRISMA® guidelines and the Cochrane Manual. The study includes a comprehensive database search, well-defined PICOS® criteria, an evaluation of methodological quality, and an analysis of the risk of bias, ensuring rigor and reliability in the findings.

Several limitations should be considered: the study only includes research from the past 15 years and is limited to English and Spanish sources; the topic has not been extensively researched, limiting the available data; and the heterogeneity of the included studies in terms of sample size, study design, and analyzed variables prevents broad generalization of results. Publication bias may be present, as most of the selected studies report positive outcomes, making them more likely to be published than those with negative or inconclusive results.

Among some of the future research lines, we can highlight the possibility of conducting comparative studies on different coach training programs to assess their impact; developing an e-learning or blended learning model tailored to the needs of basketball coaches and players; analyzing the use and benefits of new technologies in basketball coach education and training.

## 5 Practical applications

The findings of this study will support the design of standardized training programs that ensure the acquisition of essential knowledge, skills, and competencies for effective coaching. These programs will be adapted based on competitive level and age while considering the various aspects involved in basketball player development. Additionally, the study emphasizes the importance of implementing evidence-based teaching and assessment methodologies, incorporating e-learning platforms and digital technologies to facilitate active and meaningful learning.

## 6 Conclusion

There is a pressing need for basketball coach training programs that move beyond traditional models and adapt to modern coaching demands. These programs should integrate technical-tactical, physical, and psychological knowledge while fostering practical skill development through hands-on experience.

Furthermore, current training offerings should be complemented by more specialized and comprehensive continuing education, emphasizing learning construction and understanding to support the professional development of basketball coaches. This includes updating and enhancing skills within digital and informal learning environments.

Blended learning emerges as the most effective training approach, combining the autonomy of e-learning with on-court practical experience—both of which are essential for well-rounded coach education.

## Data Availability

The original contributions presented in the study are included in the article/Supplementary Material, further inquiries can be directed to the corresponding author.
